# Acute effects of posture on intraocular pressure

**DOI:** 10.1371/journal.pone.0226915

**Published:** 2020-02-06

**Authors:** Emily S. Nelson, Jerry G. Myers, Beth E. Lewandowski, C. Ross Ethier, Brian C. Samuels

**Affiliations:** 1 NASA Glenn Research Center, Cleveland, OH, United States of America; 2 NASA Glenn Research Center, Cleveland, OH, United States of America; 3 Wallace H. Coulter Dept of Biomedical Engineering, Georgia Institute of Technology and Emory University, Atlanta, GA, United States of America; 4 Dept of Ophthalmology, University of Alabama at Birmingham, Birmingham, AL, United States of America; Cardiff University, UNITED KINGDOM

## Abstract

Many experiments have documented the response of intraocular pressure (IOP) to postural change. External forces caused by gravitational orientation change produce a dynamic response that is encountered every day during normal activities. Tilting the body at a small downward angle is also relevant to studying the effects of hypogravity (spaceflight), including ocular changes. We examined data from 36 independent datasets from 30 articles on IOP response to postural change, representing a total population of 821 subjects (≥1173 eyes) with widely varying initial and final postures. We confirmed that IOP was well predicted by a simple quantity, namely the hydrostatic pressure at the level of the eye, although the dependence was complex (nonlinear). Our results show that posturally induced IOP change can be explained by hydrostatic forcing plus an autoregulatory contribution that is dependent on hydrostatic effects. This study represents data from thousands of IOP measurements and provides insight for future studies that consider postural change in relation to ocular physiology, intraocular pressure, ocular blood flow and aqueous humor dynamics.

## Introduction

The phenomenon of pressure elevation in the eyes and surrounding blood vessels after changing posture from upright to supine has been studied for over 50 years. [1–3] This alteration of IOP has been attributed to changes in blood pressure within the episcleral veins. [4–6] Studies have described rapid changes in IOP which are contemporaneous with tilting, [7–9] which can be explained purely by hydrostatic blood pressure changes. [10] After this very acute response, the IOP then undergoes smaller, more gradual alteration as the eye comes to a new equilibrium state over tens of minutes to an hour, [8, 11–14] which can be attributed to aqueous humor dynamics and ocular hemodynamics. [10] Thus, the IOP temporal profile after postural change is multiphasic in nature. [8, 10, 12, 14]

Despite the many studies on IOP and postural change in the literature, it appears that there has been no attempt to synthesize these findings. We undertook such an analysis to uncover relationships between IOP and posture that might not be evident from any single study. To minimize uncertainties arising from the dynamic nature of the process, we sought studies that allowed sufficient time for IOP equilibration at the new posture (see Methods).

In previous work, we created a numerical model of the IOP response to acute gravitational change that incorporated the effects of dynamically changing aqueous humor and ocular blood volumes, local arterial and venous pressures, and intracranial pressure. [10] This biophysical model compared very well to experimental data in several acute gravitational scenarios, including head-down tilt studies of duration 5 to 21 minutes. Although the predictions remained within experimental uncertainty during the experimental time frame, the experimental data appeared to plateau earlier than the simulated data. Beyond the experimental time frame, the simulations exhibited a slow, continuous increase in IOP and reached steady state after about an hour of simulated time. The IOP at the end of the experiment tended to be somewhat lower than the steady-state numerically generated value. We speculated that there may be a physiological regulatory mechanism affecting IOP from about 10 minutes to an hour after the postural change that was not included in our numerical model. This investigation began as an attempt to discern the extent of such physiological regulation after the most acute stage of postural change.

## Methods

We identified 36 independent datasets in 30 articles on IOP response to postural change that fit the criteria defined below. [6–8, 11–37] They included a variety of scenarios, such as upright to supine posture and vice versa, upright to head-down tilt (HDT) or head-up tilt (HUT), supine to HDT or HUT, and cycling between positive and negative tilt angles. We included studies with as few as 3 or as many as 151 eyes. [25, 38] When available, we included in Table 1 other potentially relevant data such as gender, height, weight, age, and mean arterial pressure (MAP). The latter was found to exhibit a considerable impact on IOP in prior work. [10] When results from both eyes were documented or the data was presented by subgroups, we averaged the results. When necessary, we converted standard errors of the mean and confidence intervals to standard deviations for consistency. In some cases, we estimated the standard deviations visually or by digitization. When not specified, we assumed that only one eye per subject was examined, leading to a net population of 821 subjects and ≥1173 eyes.

**Table 1:** Studies included in this investigation.

Authors	n_sbj_	n_eyes_	n_F_	n_M_	Height (cm)	Weight (kg)	Age (yrs)	MAP_0_ (mmHg)	MAP_f_ (mmHg)	θ_0_ (deg)	P_h,O_	IOP_0_ (mmHg)	θ_f_(deg)	P_h,f_	t (min)	IOP(t)
Anderson et al. (2017)^11^	10	10	5	5			31±7	91±8	89±9	90	1	10.7±1.6	0	0.00	1	12.2±1.7
															15	12.7±2.2
															30	12.6±3.1
															45	12.3±3.3
															60	12.2±3.0
Balasubramanian et al. (2017)^26^	20	20	8	12			33±2			90	1	16.7±2.5	0	0.00	10	18.8±3.9
													-15	-0.26	20	20.3±3.9
Barkana (2014)^17^ (V)	19	38	9	10			33.0±12.4			90	1	16.6±3.2	0	0.00	15	20.8±2.8
															45	19.7±2.4
Baskaran et al. (2006)^7^	75	75	51	24			48.9±13.7			90	1	14.2±2.9	-90	-1.00	1	29.3±4.4
															5	30.1±4.8
Carlson et al. (1987)^27^ (V, n_sbj =_ 10,20)	11	11					14–47			0	0		15	0.26	60	14.5±1.2
										0	0		-15	-0.26	60	16.8±1.4
	10	10					14–47			15	0.259	13.5±0.3	-15	-0.26	60	15.7±0.5
	20	20					14–47			50	0.766	13.6±0.3	-50	-0.77	30	25.0±0.7
Chiquet et al. (2003)^28^	25	50	25	0			27 (median)			90	1	16.1±3.6	0	0.00	1	18.3±3.6
															3	17.0±3.0
															10	18.0±3.8
Draeger et al. (1986)^12^ (V)	20	20					23–41	100	0	90	1	11.6±2.1	-90	-1.00	6	34.3±3.5
										-90	-1	34.3±3.5	90	1.00	6	11.6±1.8
	10	10	0	10			same age			0	0	15.6±2.5	-10	-0.17	0.5	18.6±2.7
															15	23.6±2.2
															90	16.9±2.7
Eklund et al. (2016)^6^ (V)	11	22	8	3			46±10			0	0	17.2±1.8	90	1.00	25	14.5±2.3
Friberg and Weinreb (1985)^8^	16	32	9	7	173±8		30±5			90	1	14.1±2.8	-90	-1.00	5	35.6±4.0
										-90	-1	35.6±4.0	90	1.00	5	14.1±4.0
Friberg and Weinreb (1987)^29^	11	22								0	0	19.0±3.6	-90	-1.00	1 to 30	36.9±4.0
Jain and Marmion (1976}^30^	20	40	20	0			20.75 (15–29)			90	1	15.65±1.12	0	0.00	5	16.88±3.47
	76	151					61.72 (30–85)			90	1	17.3±3.07	0	0.00	2	20.01±3.47
															5	19.75±3.47
James and Smith (1991)^31^	28	28	23	5			51.3 (29–73)	98±12	90±11	90	1	17.6±3.17	0	0.00	15	19.8±3.2
Katsanos et al. (2017)^32^	21	21					71.4±5.6	1003±10.6	97.5±10.2	90	1	13.6±1.9	-20	-0.34	10	14.4±2.6
Laurie et al. (2017)^33^	8	16	0	8	181 (170–193)	86 (73–95)	35(25–49)	89 (82–96)	87 (80–94)	90	1	15.0±2.2	-6	-0.10	5	15.7±2.6
Linden et al. (2017)^34^	11	11	8	3	165	77	47(30–59)	103		0	0	17.2±1.8	90	1.00	15	14.5±2.3
Linder et al. (1987)^35^	3	3								90	1	13	-90	-1.00	1.5	36.6
	10	10								90	1	11.8	-6	-0.10	1	15.5
															30	*15*
															60	14
															90	14.9
Linder et al. (1988)^13^ (V)	10	20					18–36			90	1	12.2±2.2	-6	-0.10	1	15.5±2.2
															30	15.7±2.2
															60	14.6±2.2
															90	14.7±2.2
Longo et al. (2004)^36^	11	11	4	7			32±13	96±10	95±8	90	1	13±1	-8	-0.14	5	17±2
Macias et al. (2015)^37^	25	25	11	14			36			0	0	21.0±0.4	90	1.00	5	17.8±0.4
													-15	-0.26	5	21.9±0.5
Marshall-Goebel et al. (2017)^38^	9	9	0	9			25.0±2.4	73.2±10.8	82.0±15.3	0	0	15.3±1.2	-6	-0.10	210	16.3±0.9
										0	0	15.7±0.9	-12	-0.21	210	17.9±1.2
								71±10	84.3±8.1	0	0	15.8±1.2	-18	-0.31	210	18.7±1.2
	16	16	8	8			26.6±3.7			0	0	14.8±0.4	12	0.21	5	14.4±1.2
								75.3±16.4	81.8±20.4	0	0	14.7±0.4	-12	-0.21	5	15.7±1.6
								71±10	84.3±10.8	0	0	14.7±0.4	-18	-0.31	5	16.5±1.2
										0	0	14.6±1.2	-24	-0.41	5	18.4±1.6
Selvadurai et al. (2010)^39^	21	42	9	12			31.6±6.9			90	1	17.8±1.9	0	0.00	5	19.9±1.7
Seo et al. (2015)^40^	17	34	6	11			28.3±2.7			90	1	13.0±2.0	0	0.00	5	14.8±1.9
Shinojima et al. (2012)^41^	9	9	2	7	169±3	60±4	34±2	81±9	74±6	90	1	14±3	-10	-0.17	30	21.0±4.5
Singh et al. (1986)^42^	58	58	29	29						90	1	14.14±3.56	0	0.00	5	15.96±4.03
Singleton et al. (2003)^43^	11	22	5	6			60.8±10.9	91±9	95±9	0	0	16±3	90	1.00	10	15±2
Souza et al. (1987)^15^ (V)	20	40	10	10	169.7	66.0	29.2,20–40	101±9	114±17	90	1	12.0±3.2	-90	-1.00	5	28.29±3.80
Sultan and Blondeau (2003)^44^ (V)	74	74	28	46			21.4,77.6	94	88.5	90	1	15.0±2.7	0	0.00	15	16.9±3.1
Ventura et al. (2014)^45^	16	16					49.8±12.3			90	1	14.1±2.3	-10	-0.17	8	17.3±4.1
Xu et al. (2010)^14^	65	129					22–23	88.1	86.7	0	0	15.5±4.5	-15	-0.26	1	18.5±4.5
															6	18.6±4.5
															11	18.3±4.5
															16	18.1±4.5
															21	17.8±4.5
Yeon et al. (2014)^46^	24	48	11	13			26.7±1.3			90	1	13.4±2.2	0	0.00	10	16.9±23

Columns indicate the number of subjects (n_sbj_), number of eyes studied (n_eyes_), number of females (n_F_) and males (n_M_), height, weight, age, mean arterial pressure (MAP), tilt angle (θ), nondimensional hydrostatic pressure (ph), intraocular pressure (IOP), measurement time (t) relative to commencement of postural change at initial (subscript 0) and final (subscript f) times. Studies reserved for validation are denoted by “V”.

### Hydrostatic pressure and postural change

**Fig 1:**
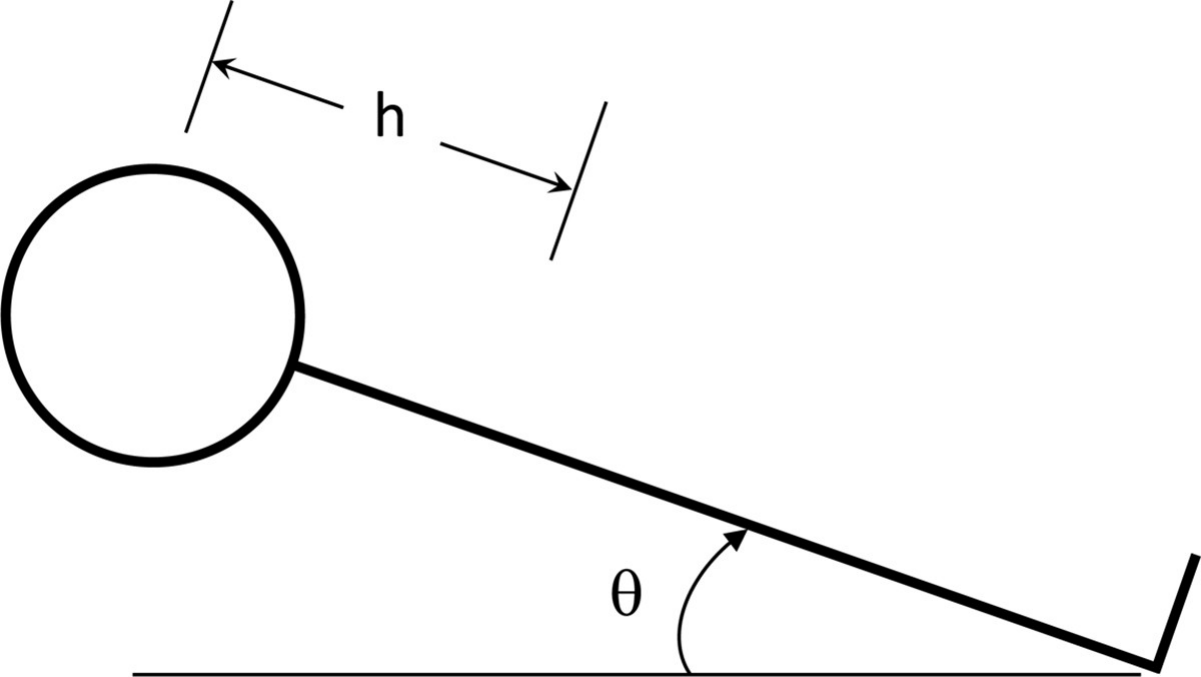
Schematic of body position as a function of tilt angle θ. Height (h) is the distance between the aortic root and the eyes along the body axis.

We took the supine position as the reference posture, (i.e. a tilt angle θ of zero in Fig 1). With this nomenclature, an upright posture corresponds to a tilt angle θ of +90°, while θ = -90° represents an inverted posture. In this study, we assumed that there was no IOP difference between standing and seated postures, since the reported difference is small in comparison to the other sources of error. [34]

At any point in time, the blood pressure predicted by simple hydrostatics (hydrostatic pressure, ${\hat{p}}_{h}$) at the level of the eye is ρgh·sin θ, where ρ is the density of blood, g is the acceleration of gravity, h is the distance between the eye and the aortic root (the “hydrostatic indifference point”, taken as a datum), as shown in Fig 1. We defined a nondimensional physical stimulus p_h_ as the hydrostatic contribution to blood pressure at the level of the eye normalized by ρgh, or simply *p_h_* = sin θ.

### Inclusion criteria

We examined over 100 studies that measured the response of intraocular pressure to postural change in healthy individuals. Table 1 identifies the datasets included in this study. We used a combination of PubMed searches and Google Scholar searches, followed by examination of studies cited by the original group of papers, etc. The criteria for evaluating whether to include a study were:

#### Reporting requirements

We required each dataset to have a well-documented reference position, experimental protocol, and the time at which the measurements were taken relative to the onset of the new posture.

#### Anesthetic usage

The response of IOP to ocular volume change, e.g., during saline injection studies, is different when retrobulbar anesthesia is used as compared to topical anesthesia. [39] Head-down tilt (HDT) also causes fluid volume change in the eye, although it is driven by hydrostatics and physiological factors rather than direct intraocular fluid injection. We therefore only considered studies that used at most a topical anesthetic before IOP measurements were taken.

#### Equilibration time

The time required by IOP to reach its equilibrium value at a new posture may vary with tilt angle. [8, 12–14] Some studies indicate that IOP may still be changing at 45 minutes, [12, 13, 16] although most studies allow for a much shorter time period in the range of 2 to 5 minutes. We therefore discarded studies that subjected their cohort to a single postural change for durations of <5 minutes. We did not include studies with protocols that specified a sequence of tilt angles, unless: (a) an IOP time series was taken to ensure that equilibration had been achieved at each tilt angle (θ); or (b) θ changed in a monotonic fashion from the start to conclusion of the experiment. Prior work showed that the IOP response to postural change is initially driven only by the gravitationally driven blood pressure change at the eye. [10] For case (b), we reasoned that each change in tilt angle produced incremental quasi-equilibrium states that reached their full equilibrium value at the final tilt angle. In this case, we used the baseline reading and the reading at the final tilt angle, provided that the net time from initial to final posture was ≥5 min.

#### Measurement error

Since the difference in IOP (ΔIOP) for small postural changes may be as small as 1 to 2 mmHg, measurement error is important because it may be comparable to or larger than the IOP changes we would like to capture. In general, inter-device IOP measurements exhibit a measurement difference in the range of 0.5 to 2mmHg on the same eyes under the same conditions. [16, 40–44] In one case, the inter-device agreement became better at 45 minutes as opposed to 15 minutes, [16] highlighting the importance of allowing sufficient equilibration time. One study in this work presents data using four instruments; [16] we used only the pneumatonometer data in this case. Measurement accuracy also depends on globe properties such as central corneal thickness, and on device calibration, patient stillness and hydration level, and operator experience, [12, 16, 40–43, 45] although we did not control for these factors. Due to the significant dependence of IOP on posture, we did not include studies in which the subject’s posture had to change in order to take the IOP measurement. Handheld tonometers are attractive as measurement devices in this regard, because they can be operated at a variety of angles, although their performance in terms of accuracy is mixed. [16, 40, 46, 47] Although there are mixed findings on the effects of pillow usage, [20, 30, 37] where IOP was measured without the use of pillows when they were available. However, we included two studies that only contained data with use of pillows. (James and Smith, 1991; Shinojima et al., 2012) [21, 31]

We randomly chose 8 datasets from 7 articles for validation (denoted by “V” in Table 1), and used the remainder for the curve fit. When multiple studies existed at a specific p_h_, we weighted each study by the ratio of its number of eyes to the total number of eyes for all of the studies at that particular p_h_.

### Curve fitting and statistics

The mean square error for the curve fit is $MSE=\sum r_{i}^{2}/(n-n_{p}-1)$, where the residual r_i_ is the difference between experimental and predicted response, n is the sample size, and n_p_ is the number of fitted parameters. We used a function finder at http://www.zunzun.com to determine that an exponential curve fit was the best choice from the standpoint of minimizing MSE. We used MATLAB (Mathworks, Inc., Natick, MA) to calculate the exponential curve fit and develop additional statistics. To confirm that the curve fit was independent of our randomization, we repeated the curve-fitting procedure twice, each time choosing a different random sample of 28 datasets. The resulting IOPs generated by the three curve fits varied from one another by less than 3% at the most extreme.

**Fig 2:**
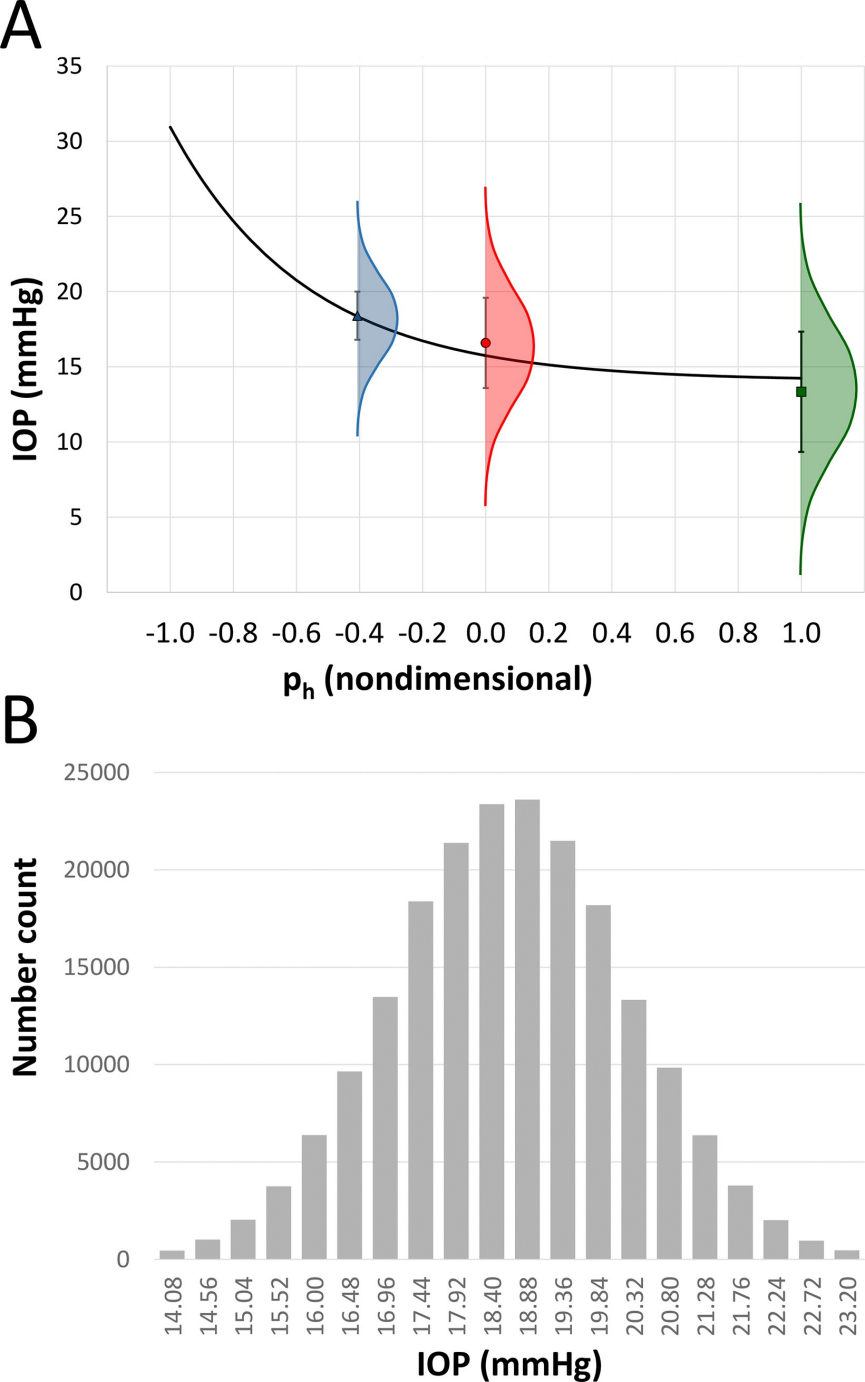
Development of simulated IOP datasets from Latin hypercube simulation. (A) Schematic of the relationship of experimental mean data (symbols) and standard deviations (error bars) to Gaussian distributions. (B) Histogram representing the IOP distribution in 300,000 simulated datasets for an experimental data point at left (p_h_ = -0.41).

The experiments themselves often had substantial experimental uncertainty, as shown in Table 1, and represented graphically in Fig 2. We wanted to show a confidence metric that took into account that experimental uncertainty. Fig 2A shows some representative data points used in the curve fit and displays the mean value, standard deviation and a schematic of the corresponding Gaussian distribution.

A Latin hypercube simulation (LHS) delivers a set of values that fall within a specified distribution. [48] For the curve fit, we had 61 measurements of mean IOPs and standard deviations at fixed p_h_ values. From that data, we used an LHS simulation in MATLAB to calculate 300,000 independent sets of IOPs, using 3 times the standard deviation to specify cutoff values. Fig 2B shows the IOP distribution as calculated by LHS for one of the 61 experimental measurements.

We ran each simulated IOP set through the curve-fitting procedure, as described above. Across the range of p_h_, we then calculated the maximum and minimum predicted IOP values over all of the LHS curves, which will be shown below.

### Numerical simulation of IOP response to postural change

In prior work, we subjected a 5-compartment lumped-parameter model of the eye to acute gravitational changes to calculate volume, pressure and flow interactions among compartments representing the globe, arterial and venous ocular blood, aqueous humor, and the retrobulbar subarachnoid space. [10] The model included hydrostatic effects on ocular pressures. It did not include explicit regulatory functions, but compliance functions may have had some implicit regulation since they were derived from empirical data.

In virtual head-down tilt, the IOP responded immediately to gravitational changes in a linear fashion during the transition between initial and final postures, as is consistent with experimental data. The gravitational forcing also caused abrupt changes to the local ocular arterial and venous pressures. After the final posture was attained, a more gradual increase in IOP occurred over the course of ten minutes to an hour as the aqueous and blood volumes adjusted to the newly imposed ocular pressures.

We used the steady-state IOP values obtained in these simulations to approximate the hydrostatic contribution to IOP.

## Results

**Fig 3:**
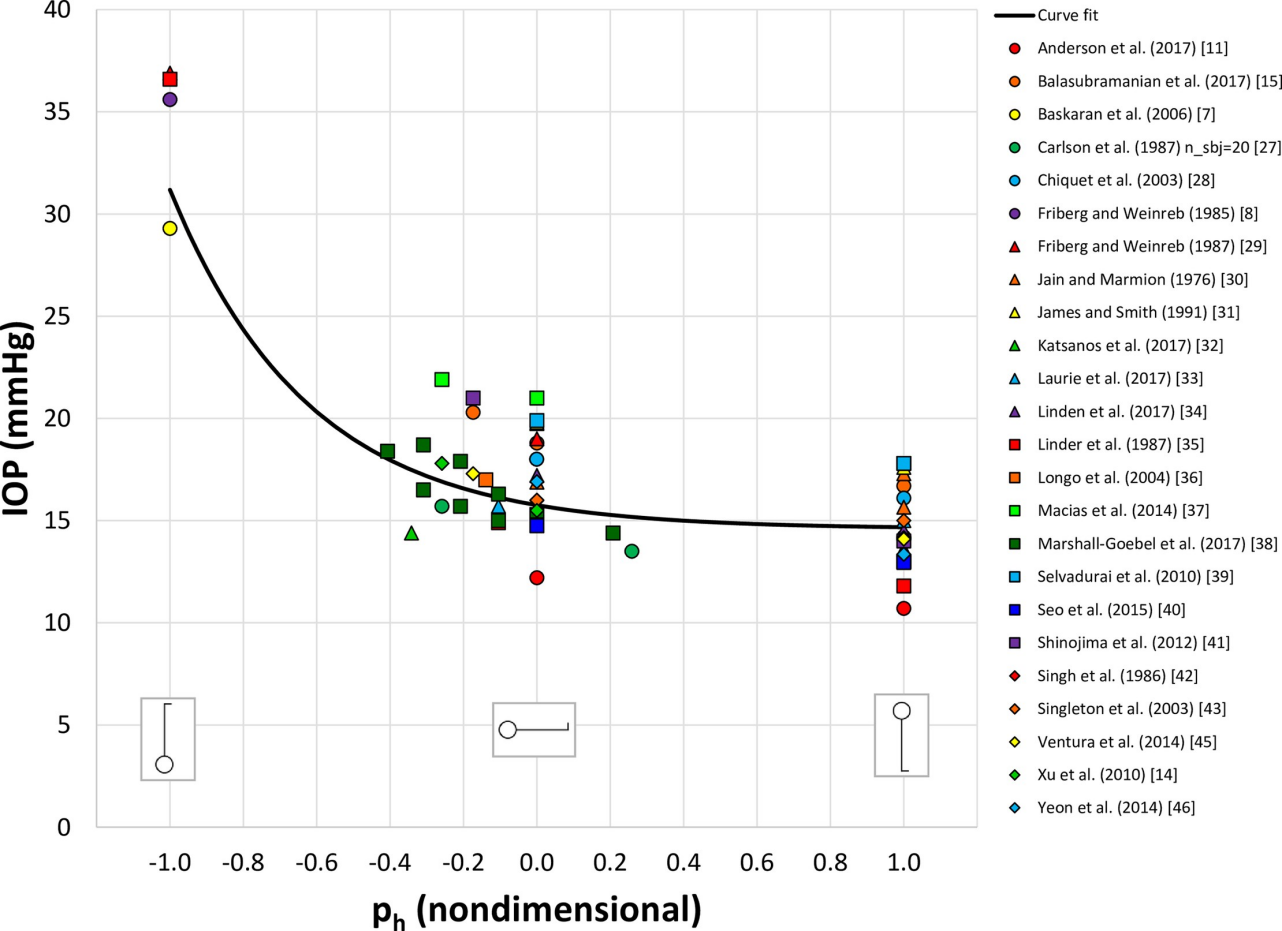
Relationship between the equilibrium intraocular pressure (IOP) and the “hydrostatic forcing function”, p_h_. Markers indicate experimental measurements from 28 studies on 657 subjects (≥949 eyes). Solid line is the curve fit, as defined by Eq (1). When multiple values of IOP were available at a specific p_h_, the curve fit weighted the data by the ratio of the number of subjects in the study to the total number of subjects at that p_h_.

After examining the literature, we found 36 datasets from 30 articles [6–9, 11–37] that fit our inclusion criteria (Table 1). We reserved 8 randomly selected studies for validation and used data from the remaining 28 studies for the curve fit (see Methods for details). We found that the best fit for IOP was:

$$IOP=1.16\;\exp\left(-2.66\;p_{h}\right)+14.6$$ (1)

where IOP is expressed in mmHg, shown in Fig 3. The data at the far left (p_h_ = -1.0) come from postural inversion while the data at the far right (p_h_ = +1.0) represent the upright posture. For reference, a linear fit of the data yielded a mean squared error (MSE) of 15.51 mmHg^2^ while the exponential curve fit exhibited a significantly smaller MSE of 0.85 mmHg^2^. The graph of the residual r_i_ vs. p_h_ for the exponential fit shows a nearly random distribution about the mean residual, while the linear fit exhibited marked curvature, an indicator of the presence of nonlinearity in the source data. [49]

**Fig 4:**
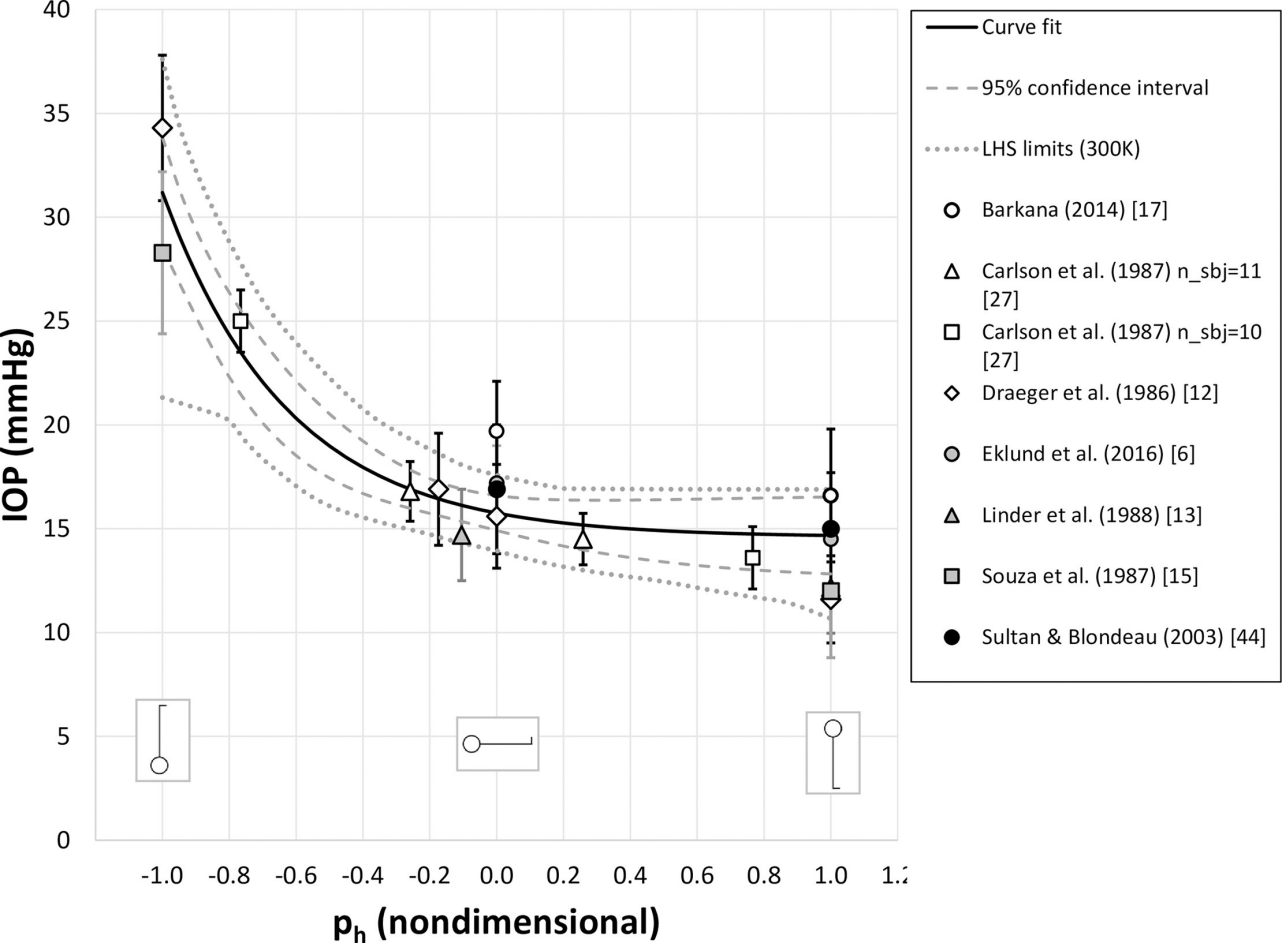
Curve fit (black line; Eq 1) compared against 8 validation datasets (symbols), plotted with standard deviations. Dashed line represents the 95% confidence interval of the curve fit. Dotted line is derived from the extrema of curve fits of 300,000 simulated sets of IOPs, which were generated by a Latin hypercube simulation (LHS) using experimental means and standard deviations.

We reproduced the curve fit from Eq (1) in Fig 4 (solid line) and included data from the 8 validation datasets (symbols), plotted with their respective standard deviations. The dashed lines are the 95% confidence intervals, while the dotted lines represent the extrema predicted by a set of 300,000 curve fits. For this metric, each simulated dataset was generated by a Latin hypercube simulation based on experimental means and standard deviations (see Methods). As expected, nearly all of the experimental means fell inside the 95% confidence interval (CI) to within experimental error. The study with the most significant departure was that of Barkana (2014) who made comparable IOP measurements using four different instruments. To enhance rigor and ensure statistically independent data sets we chose *a priori* to use only one instrument’s measurements for this work (the pneumatonometer).Had we chosen to use their GAT, Tonopen or HA-2 measurements, which were all lower than the pneumatonometer measurements, the Barkana (2014) datapoint would have fallen within the 95% confidence interval.

The IOP values from experiments with under 5 minutes for equilibration, which were not used in the curve fit, were more likely to fall outside of the LHS-derived bounds (data not shown).

**Fig 5:**
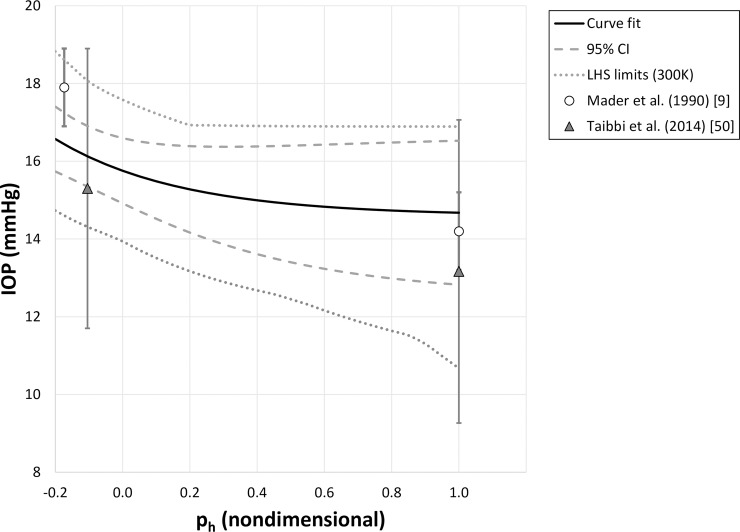
Curve fit (black line), 95% confidence interval to the curve fit (dashed) and extrema in LHS simulations (dotted) compared against experimental means (symbols) and standard deviations from baseline conditions (upright, ph = 1.0) and after 2 days of 10° HDT [9] or 10 days of 6° HDT [50].

Our curve fit was not designed to be accurate at long times (many hours to days) after postural change, since there may be additional factors that may come into play, such as changes to aqueous formation rate. Consequently, we did not include sleep studies in our curve-fitting data set. However, for interest, we compared Eq (1) against two studies of IOP change during long-duration HDT conducted as a terrestrial microgravity analog.[9, 50] The experimental data show good agreement with the curve fit (Fig 5), suggesting that posturally driven IOP changes are still an important factor after two or more days of HDT.

**Fig 6:**
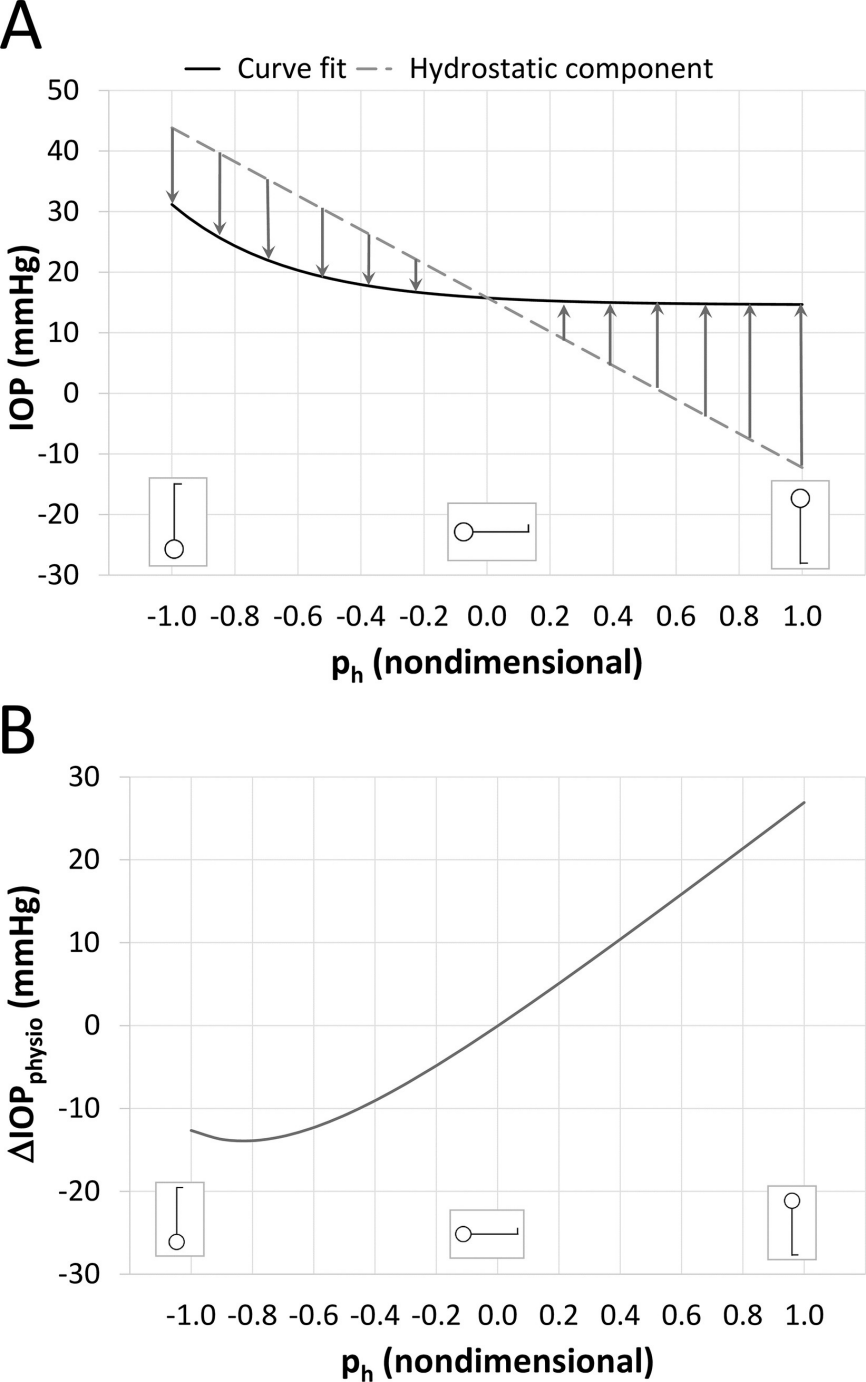
Representation of the factors affecting IOP in postural change. (A) The theoretical IOP resulting from hydrostatics alone (dashed line) needs to be adjusted by physiologic processes (arrows) to match the observed IOP after postural change (solid line). (B) The net physiologic regulation (ΔIOPphysio) is defined as the difference between IOP derived from hydrostatics and the IOP from the curve fit.

The relationship between IOP and p_h_ would be linear if hydrostatic effects alone were responsible for the IOP response. We conclude that the observed IOP after postural change is a combination of both hydrostatic and physiologic effects, and that the departure from linearity can be considered as the net effect of physiologic processes. In Fig 6A, the curve fit (solid line) is shown with a calculation of IOP using the straightforward application of hydrostatic forces (dashed line). The difference between the two represent the IOP change necessary to bring the IOP from the hydrostatic value to the observed value (arrows). We denote this difference as the physiologic component of IOP change, ΔIOP_physio_, which is shown more directly in Fig 6B, and is the net result of all physiologic effects, whether they are local or systemic, active or passive.

## Discussion

The major finding of this study is that acute changes in IOP due to postural alterations can be “universally” predicted by a simple equation in which the only dependent variable is the hydrostatically computed blood pressure at the level of the eye, consistent with the understanding that acute IOP changes result from gravitationally driven blood pressure changes. However, the predictive equation differs from that which would occur due to trivial application of hydrostatics, depicted in Fig 6A (dashed line). This important difference is almost certainly due to one or more autoregulatory processes, shown over the range of p_h_ in Fig 6B.

Since globe volume changes directly affect IOP, [10, 39, 51] the relevant regulatory mechanism(s) must ultimately involve a net loss or gain of fluid within the globe through a transient alteration of the inflow/outflow of ocular blood and/or aqueous humor. Only a small quantity of fluid needs to be involved since the addition of 1 μL of ocular fluid results in an IOP increase of about 1 mmHg. [51] It is quite possible that our current experimental and imaging techniques do not have the resolution to resolve the expected small volume changes. Data from previous studies on this subject suggest that alterations in ocular blood or aqueous equilibrium are caused by postural change, [17, 31] but validation of Fig 6 would require a better accounting of net fluid volume response in the globe. Hopefully the recent advances in the field of ophthalmic imaging will provide an opportunity to address this question more fully.

The framework presented here permits a comparison of independent experiments with differing baseline or final postures by providing a simple mathematical expression that identifies an IOP that corresponds with tilt angle regardless of baseline posture. This is consistent with the literature: studies that have examined both positive and negative changes in θ report that IOP is equivalent when approached from either direction as long as sufficient equilibration time is given. [7, 8, 12, 17]

The curve fit introduced here may influence future experimental designs and protocols by allowing an estimation of the IOP change that must be resolved during postural change. This also suggests that to capture effects that are not dominated by posture, sleep studies could choose to subtract each subject’s daytime supine IOP from their supine nocturnal measurements to separate out and more precisely determine the impact of the more subtle non-posturally driven physiological factors that arise during the night.

As another example, this curve fit can be used to design a tilt protocol based on desired IOP response. Many studies examine the physiological effects of extended HDT as a means of simulating microgravity on earth, with upright or supine posture representing pre-launch conditions, and the HDT position as the pseudo-microgravity challenge. [12, 23, 25, 27] However, these protocols do not explicitly account for the significant gravitational changes that are a result of launch and insertion into microgravity. On average, astronauts undergo a ΔIOP = 6.3 mmHg [52] by the time they reach orbital altitude after about 10 minutes of launch accelerations. For a virtual astronaut, the baseline supine IOP is 15.8 mmHg according to the curve fit. To produce an IOP rise of 6.3 mmHg, the subject could be tilted to p_h_ = -0.7 (θ = -45°) for 10 minutes to expose the eye to launch-like stresses, which could then be followed by the pseudo-microgravity phase.

This analysis is subject to uncertainties inherent to the studies that we examined and the challenges of aggregating such data. There is an unknown level of uncertainty from inter-device, inter-operator, and intra-device measurement errors, variations in experimental protocols and populations, and whether or not a complete IOP equilibrium was achieved. Ideally, we would have used data from the same research group on the same subjects using the same protocols and measurement devices, which would have improved the curve fit accuracy. While this study included significant variability in all of those factors, reduction of the data set to a single study group, experimental design, and instrument would have significantly reduced our statistical power and the confidence bounds of the curve fit. Despite the breadth of approaches used by the researchers aggregated in this study, there is good general agreement of the curve fit with the experimental data. Another limitation of this study is that it is not directly applicable to the prone posture. IOP in the prone position is known to be higher than that of supine posture, and the biomechanics are more complex. [11, 53] A similar analysis might well find a comparable relationship between IOP and p_h_ for postural change involving the prone position.

## Supporting information

S1 Data Included are the raw data for studies included in this manuscript. Studies are divided by row with (V) denoting studies reserved for the validation experiments. The columns indicate the number of subjects (nsbj), number of eyes studied (neyes), number of females (nF) and males (nM), height, weight, age, mean arterial pressure (MAP), tilt angle (θ), nondimensional hydrostatic pressure (ph), intraocular pressure (IOP), measurement time (t) relative to commencement of postural change at initial (subscript 0) and final (subscript f) times.
(XLSX)

## References

[1] Galin MA, Mc IJ, Magruder GB. Influence of position on intraocular pressure. Am J Ophthalmol. 1963;55:720–3. Epub 1963/04/01. . 13960329

[2] Tarkkanen A, Leikola J. Postural variations of the intraocular pressure as measured with the Mackay-Marg tonometer. Acta Ophthalmol (Copenh). 1967;45(4):569–75. 10.1111/j.1755-3768.1967.tb06523.x . 6072270

[3] Linner E, Rickenbach C, Werner H. Comparative measurements of the pressure in the aqueous veins and the conjunctival veins using different methods. Acta Ophthalmol (Copenh). 1950;28(4):469–78. Epub 1950/01/01. 10.1111/j.1755-3768.1950.tb00002.x . 14846559

[4] Thomassen TL. The safety valve of the eye. Acta Ophthalmol Scand. 1949;27(3):413–22.

[5] Hinghofer-Szalkay H., Gravity the hydrostatic indifference concept and the cardiovascular system. Eur J Appl Physiol. 2011;111(2):163–74. Epub 2010/09/22. 10.1007/s00421-010-1646-9 . 20857139

[6] Eklund A, Johannesson G, Johansson E, Holmlund P, Qvarlander S, Ambarki K, et al The pressure difference between eye and brain changes with posture. Ann Neurol. 2016;80(2):269–76. 10.1002/ana.24713 . 27352140

[7] Baskaran M, Raman K, Ramani KK, Roy J, Vijaya L, Badrinath SS. Intraocular pressure changes and ocular biometry during Sirsasana (headstand posture) in yoga practitioners. Ophthalmology. 2006;113(8):1327–32. 10.1016/j.ophtha.2006.02.063 . 16806478

[8] Friberg TR, Weinreb RN. Ocular manifestations of gravity inversion. JAMA. 1985;253(12):1755–7. Epub 1985/03/22. . 3974054

[9] Mader TH, Taylor GR, Hunter N, Caputo M, Meehan RT. Intraocular pressure, retinal vascular, and visual acuity changes during 48 hours of 10 degree head-down tilt Aviat Space Environ Med. 1990;61(9):810–3. WOS:A1990DW85600005. 2241746

[10] Nelson ES, Mulugeta L, Feola A, Raykin J, Myers JG, Samuels BC, et al The impact of ocular hemodynamics and intracranial pressure on intraocular pressure during acute gravitational changes. J Appl Physiol. 2017;123:352–63. 10.1152/japplphysiol.00102.2017 . 28495842 PMC5614788

[11] Anderson AP, Babu G, Swan JG, Phillips SD, Knaus DA, Toutain-Kidd CM, et al Ocular changes over 60 minutes in supine and prone postures. J Appl Physiol. 2017;123(2):415–23. 10.1152/japplphysiol.00687.2016 . 28546470 PMC6157476

[12] Draeger J, Hanke K. Postural variations of intraocular pressure—preflight experiments for the D1-mission. Ophthalmic Res. 1986;18(1):55–60. 10.1159/000265415 . 3951805

[13] Linder BJ, Trick GL, Wolf ML. Altering body position affects intraocular pressure and visual function. Invest Ophthalmol Vis Sci. 1988;29(10):1492–7. Epub 1988/10/01. . 3170121

[14] Xu X, Li L, Cao RD, Tao Y, Guo Q, Geng J, et al Intraocular pressure and ocular perfusion pressure in myopes during 21 min head-down rest. Aviat Space Environ Med. 2010;81(4):418–22. 10.3357/asem.2629.2010 WOS:000276251800010. 20377147

[15] Balasubramanian S, Tepelus T, Stenger MB, Lee SMC, Laurie SS, Liu JHK, et al Thigh cuffs as a countermeasure for ocular changes in simulated weightlessness. Ophthalmology. 2017;125(3):459–60. Epub 2017/11/21. 10.1016/j.ophtha.2017.10.023 . 29153458

[16] Barkana Y. Postural change in intraocular pressure: a comparison of measurement with a Goldmann tonometer, Tonopen XL, pneumatonometer, and HA-2. J Glaucoma. 2014;23(1):e23–8. 10.1097/IJG.0b013e3182a0762f . 24370809

[17] Carlson KH, McLaren JW, Topper JE, Brubaker RF. Effect of body position on intraocular pressure and aqueous flow. Invest Ophthalmol Vis Sci. 1987;28(8):1346–52. Epub 1987/08/01. . 3610552

[18] Chiquet C, Custaud MA, Le Traon AP, Millet C, Gharib C, Denis P. Changes in intraocular pressure during prolonged (7-day) head-down tilt bedrest. J Glaucoma. 2003;12(3):204–8. Epub 2003/06/05. 10.1097/00061198-200306000-00004 . 12782836

[19] Friberg TR, Sanborn G, Weinreb RN. Intraocular and episcleral venous pressure increase during inverted posture. Am J Ophthalmol. 1987;103(4):523–6. Epub 1987/04/15. 10.1016/s0002-9394(14)74275-8 . 3565513

[20] Jain MR, Marmion VJ. Rapid pneumatic and Mackey-Marg applanation tonometry to evaluate the postural effect on intraocular pressure. Br J Ophthalmol. 1976;60(10):687–93. Epub 1976/10/01. 10.1136/bjo.60.10.687 1009040 PMC1042810

[21] James CB, Smith SE. The effect of posture on the intraocular pressure and pulsatile ocular blood flow in patients with non-arteritic anterior ischaemic optic neuropathy. Eye (Lond). 1991;5 (Pt 3):309–14. Epub 1991/01/01. 10.1038/eye.1991.49 . 1955053

[22] Katsanos A, Dastiridou AI, Quaranta L, Rulli E, Riva I, Dimasi V, et al The effect of posture on intraocular pressure and systemic hemodynamic parameters in treated and untreated patients with primary open-angle glaucoma. Journal of ocular pharmacology and therapeutics: the official journal of the Association for Ocular Pharmacology and Therapeutics. 2017;33(8):598–603. Epub 2017/08/17. 10.1089/jop.2017.0030 . 28813622

[23] Laurie SS, Vizzeri G, Taibbi G, Ferguson CR, Hu X, Lee SMC, et al Effects of short-term mild hypercapnia during head-down tilt on intracranial pressure and ocular structures in healthy human subjects. Physiol Rep. 2017;5(11). 10.14814/phy2.13302 28611153 PMC5471441

[24] Linden C, Qvarlander S, Johannesson G, Johansson E, Ostlund F, Malm J, et al Normal-tension glaucoma has normal intracranial pressure: A prospective study of intracranial pressure and intraocular pressure in different body positions. Ophthalmology. 2017;125(3):361–8. Epub 2017/11/04. 10.1016/j.ophtha.2017.09.022 . 29096996

[25] Linder BJ, Trick GL. Simulation of spaceflight with whole-body head-down tilt: influence on intraocular pressure and retinocortical processing. Aviat Space Environ Med. 1987;58(9 Pt 2):A139–42. Epub 1987/09/01. . 3675480

[26] Longo A, Geiser MH, Riva CE. Posture changes and subfoveal choroidal blood flow. Invest Ophthalmol Vis Sci. 2004;45(2):546–51. Epub 2004/01/28. 10.1167/iovs.03-0757 . 14744897

[27] Macias BR, Liu JH, Grande-Gutierrez N, Hargens AR. Intraocular and intracranial pressures during head-down tilt with lower body negative pressure. Aviat Space Environ Med. 2015;86(1):3–7. 10.3357/AMHP.4044.2015 . 25565526

[28] Marshall-Goebel K, Mulder E, Bershad E, Laing C, Eklund A, Malm J, et al Intracranial and intraocular pressure during various degrees of head-down tilt. Aerosp Med Hum Perform. 2017;88(1):10–6. 10.3357/AMHP.4653.2017 . 28061916

[29] Selvadurai D, Hodge D, Sit AJ. Aqueous humor outflow facility by tonography does not change with body position. Invest Ophthalmol Vis Sci. 2010;51(3):1453–7. Epub 2009/12/05. 10.1167/iovs.09-4058 iovs.09-4058 [pii]. . 19959645

[30] Seo H, Yoo C, Lee TE, Lin S, Kim YY. Head position and intraocular pressure in the lateral decubitus position. Optom Vis Sci. 2015;92(1):95–101. Epub 2014/12/02. 10.1097/OPX.0000000000000432 . 25437907

[31] Shinojima A, Iwasaki K, Aoki K, Ogawa Y, Yanagida R, Yuzawa M. Subfoveal choroidal thickness and foveal retinal thickness during head-down tilt. Aviat Space Environ Med. 2012;83(4):388–93. 10.3357/asem.3191.2012 WOS:000301896500002. 22462366

[32] Singh M, Sylvia Chin Suet H. Postural behaviour of intraocular pressure. Med J Malaysia. 1986;41(1):38–43. Epub 1986/03/01. . 3796346

[33] Singleton CD, Robertson D, Byrne DW, Joos KM. Effect of posture on blood and intraocular pressures in multiple system atrophy, pure autonomic failure, and baroreflex failure. Circulation. 2003;108(19):2349–54. 10.1161/01.CIR.0000097114.11038.26 . 14597588

[34] Souza SA. Cardioperipheral vascular effects of inversion on humans. Phys Ther. 1987;67(5):680–7. Epub 1987/05/01. 10.1093/ptj/67.5.680 . 3575425

[35] Sultan M, Blondeau P. Episcleral venous pressure in younger and older subjects in the sitting and supine positions. J Glaucoma. 2003;12(4):370–3. 10.1097/00061198-200308000-00013 . 12897584

[36] Ventura LM, Golubev I, Lee W, Nose I, Parel JM, Feuer WJ, et al Head-down posture induces PERG alterations in early glaucoma. J Glaucoma. 2013;22(3):255–64. Epub 2011/12/06. 10.1097/IJG.0b013e318232973b 22138883 PMC3296897

[37] Yeon DY, Yoo C, Lee TE, Park JH, Kim YY. Effects of head elevation on intraocular pressure in healthy subjects: raising bed head vs using multiple pillows. Eye (Lond). 2014;28(11):1328–33. Epub 2014/09/06. 10.1038/eye.2014.211 25190537 PMC4274296

[38] Liu JH, Sit AJ, Weinreb RN. Variation of 24-hour intraocular pressure in healthy individuals: right eye versus left eye. Ophthalmology. 2005;112(10):1670–5. Epub 2005/08/13. 10.1016/j.ophtha.2005.05.007 . 16095707

[39] Dastiridou AI, Ginis HS, De Brouwere D, Tsilimbaris MK, Pallikaris IG. Ocular rigidity, ocular pulse amplitude, and pulsatile ocular blood flow: The effect of intraocular pressure. Invest Ophthalmol Vis Sci. 2009;50(12):5718–22. 10.1167/iovs.09-3760 WOS:000272355900030. 19608534

[40] Cook JA, Botello AP, Elders A, Fathi Ali A, Azuara-Blanco A, Fraser C, et al Systematic review of the agreement of tonometers with Goldmann applanation tonometry. Ophthalmology. 2012;119(8):1552–7. 10.1016/j.ophtha.2012.02.030 . 22578443

[41] Hsu SY, Sheu MM, Hsu AH, Wu KY, Yeh JI, Tien JN, et al Comparisons of intraocular pressure measurements: Goldmann applanation tonometry, noncontact tonometry, Tono-Pen tonometry, and dynamic contour tonometry. Eye (Lond). 2009;23(7):1582–8. 10.1038/eye.2009.77 . 19407845

[42] Kotecha A, White E, Schlottmann PG, Garway-Heath DF. Intraocular pressure measurement precision with the Goldmann applanation, dynamic contour, and ocular response analyzer tonometers. Ophthalmology. 2010;117(4):730–7. Epub 2010/02/04. 10.1016/j.ophtha.2009.09.020 S0161-6420(09)01067-7 [pii]. . 20122737

[43] Tonnu PA, Ho T, Sharma K, White E, Bunce C, Garway-Heath D. A comparison of four methods of tonometry: method agreement and interobserver variability. Br J Ophthalmol. 2005;89(7):847–50. Epub 2005/06/21. 89/7/847 [pii] 10.1136/bjo.2004.056614 15965164 PMC1772716

[44] Lam AK, Wu Y-F, Wong L-W, Ho N-L. IOP variations from sitting to supine postures determined by rebound tonometer. Journal of Optometry. 2013;6:95–100.

[45] Tonnu PA, Ho T, Newson T, El Sheikh A, Sharma K, White E, et al The influence of central corneal thickness and age on intraocular pressure measured by pneumotonometry, non-contact tonometry, the Tono-Pen XL, and Goldmann applanation tonometry. Br J Ophthalmol. 2005;89(7):851–4. Epub 2005/06/21. 89/7/851 [pii] 10.1136/bjo.2004.056622 15965165 PMC1772720

[46] De Moraes CG, Prata TS, Liebmann J, Ritch R. Modalities of tonometry and their accuracy with respect to central corneal thickness and irregularities. Journal of Optometry. 2008;1:43–9.

[47] Kouchaki B, Hashemi H, Yekta A, Khabazkhoob M. Comparison of current tonometry techniques in measurement of intraocular pressure. J Curr Ophthalmol. 2017;29(2):92–7. Epub 2017/06/20. 10.1016/j.joco.2016.08.010 28626817 PMC5463014

[48] Iman RL. Latin hypercube sampling In: Balakrishnan N, Colton T, Everitt B, Piegorsch W, Ruggeri F, Teugels JL, editors. Wiley StatsRef: Statistics Reference Online 2014.

[49] Altman N, Krzywinski M. Regression diagnostics. Nature Methods. 2016;13:385 10.1038/nmeth.3854

[50] Taibbi G, Cromwell RL, Zanello SB, Yarbough PO, Ploutz-Snyder RJ, Godley BF, et al Ocular outcomes evaluation in a 14-day head-down bed rest study. Aviat Space Environ Med. 2014;85(10):983–92. 10.3357/ASEM.4055.2014 . 25245897 PMC4240225

[51] Silver DM, Geyer O. Pressure-volume relation for the living human eye. Curr Eye Res. 2000;20(2):115–20. Epub 2000/01/05. . 10617912

[52] Alexander DJ, Gibson CR, Hamilton DR, Lee SMC, Mader TH, Otto C, et al Evidence Report: Risk of spaceflight-induced intracranial hypertension and vision alterations. NASA, 2012.

[53] Anderson AP, Swan JG, Phillips SD, Knaus DA, Kattamis NT, Toutain-Kidd CM, et al Acute effects of changes to the gravitational vector on the eye. J Appl Physiol. 2016;120(8):939–46. 10.1152/japplphysiol.00730.2015 . 26662052

